# Study of Jianpi Mixture on Intestinal Microbiota of Diarrhea Irritable Bowel Syndrome Mice

**DOI:** 10.1155/2020/5241308

**Published:** 2020-04-30

**Authors:** Qiuhua Lian, Hengyue Ding, Huiping Zhu, Chuan Zhang, Song Yu, Hui Jie, Yifan Zhang, Bijian Wu, Guoqiang Liang, Guoxing Zhang, Hongwen Sun

**Affiliations:** ^1^Nanjing University of Chinese Medicine, Nanjing 210000, China; ^2^Suzhou TCM Hospital Affiliated to Nanjing University of Chinese Medicine, Suzhou 215000, China; ^3^Department of Physiology, Soochow University, Suzhou 215000, China

## Abstract

To investigate the differences in intestinal microbiota between diarrhea irritable bowel syndrome mice (IBS-D) and healthy mice and to explore the effects of Jianpi mixture on intestinal microbes' changes in IBS-D mice based on 16S rDNA sequencing analysis. 48 young ICR male mice were randomly divided into four groups (*n* = 12): (1) control group, (2) IBS-D group fed with distilled water, (3) IBS-D group fed with lactic acid bacteria compound, and (4) IBS-D group fed with Jianpi mixture for 14 days. At the end of the treatment period, 5 mice were randomly selected from each group, and then the changes in intestinal microbiota in the mice before and after treatment were analyzed by 16S rDNA high-throughput gene sequencing. Compared with the control group, the species richness and species diversity of intestinal microbiota in feces and intestinal mucosa of IBS-D mice were decreased (*P* < 0.05); IBS-D mice showed changes in composition of and in ratio of the intestinal microbiota in feces and intestinal mucosa at the level of phylum, class, order, family, genus, and species. Treatment with Jianpi mixture increased the species diversity of intestinal microbiota in IBS-D mice (*P* < 0.05) and the abundance of beneficial bacteria (*P* < 0.05) and decreased the abundance of harmful bacteria (*P* < 0.05) at the level of phylum and genus. Compared with healthy mice, the species richness and species diversity of intestinal microbiota of IBS-D mice are decreased. The intervention with Jianpi mixture can improve its diversity and regulate the equilibrium between beneficial and harmful bacteria.

## 1. Introduction

Irritable bowel syndrome (IBS) is a functional bowel disease characterized by abdominal pain and bloating discomfort accompanied by changes in bowel habits and feces characteristics. In clinic, IBS is divided into four types: diarrhea-predominant irritable bowel syndrome, constipation-predominant irritable bowel syndrome, mixed irritable bowel syndrome, and unclassified irritable bowel syndrome, and diarrhea-predominant irritable bowel syndrome (IBS-D) is its main subtype [1]. IBS-D has high incidence and significant negative impact on the quality of life and social activities of the patients. The pathogenesis of IBS-D is not completely clear, and it is mainly caused by genetic factors, gastrointestinal dysfunction, visceral hypersensitivity, intestinal inflammation, immune dysfunction, and brain-gut axis feedback abnormalities [2]. The traditional opinion is that IBS-D is a functional bowel disease, but more and more studies have revealed that the pathogenesis of IBS-D is an immune-inflammation-related disease, which is a local, sustained, low-grade inflammatory response of the intestine caused by microorganisms. As the research progression on the field, it has been explored that the intestinal microbiota is closely related to IBS-D. Microorganisms in the intestine can build intestinal barriers, help the body to digest and absorb, and synthesize various nutrients and maintain intestinal immune function, thus maintaining the balance of the ecosystem. Related reports indicate that once the dynamic balance of the intestinal microbiota is broken, resulting in imbalance of intestinal microbiota, it causes intestinal dysfunction, leading to the occurrence of IBS-D [3].

In traditional Chinese medicine (TCM), IBS-D is ascribed to “diarrhea” and “abdominal pain” according to its clinical symptom. TCM theory proposes that basic pathogenesis of diarrhea is wetness, and the main dysfunctional organ is the spleen although it exhibits intestinal symptom. Recent studies have shown that spleen-strengthening and humidifying drugs are the main approaches for IBS, especially IBS-D, in clinic [4]. It has been reported that plus Sijunzi decoction can ameliorate the main symptoms of IBS-D patients, its effect is slow but excellent, and the efficiency is stable, which is worthy of clinical application [5]. Sijunzi decoction can significantly improve the microbiota diversity in the intestine of diarrhea mice, especially for the recovery of Bifidobacterium and Lactobacillus [6]. The Jianpi mixture applied in the present study is based on Sijunzi decoction with modifications and is found to have a satisfied curative effect on the treatment of IBS-D patients.

Current research methods on the relationship between intestinal microecology and disease mainly include traditional fecal bacterial culture counting, denaturing gradient gel electrophoresis (DGGE), temperature denaturing gradient gel electrophoresis (TGGE), real-time quantitative fluorescent PCR, high-sensitivity high-throughput sequencing, and whole-genome sequencing. This study used 16SrDNA high-throughput gene sequencing technology to observe the changes in intestinal microbiota in IBS-D mice. In addition, the effect of Jianpi mixture on the intestinal microbiota of mice was observed and analyzed.

## 2. Materials and Methods

### 2.1. Experimental Animals

#### 2.1.1. Animals

Clean-grade young ICR male mice weighing 20 ± 2 g were purchased from Zhaoyan New Drug Research Center Co., Ltd., Suzhou New District, Jiangsu Province, license number: scxk (Su) 2013–0003. The light is dark and half daily, the room temperature is controlled at 23 ± 2°C, and the relative humidity is 45 to 70%. The mice were fed aseptic standard feed (purchased from Suzhou Shuangshi Experimental Animal Feed Technology Co., Ltd.), fed freely, and given water. All animal experiments were conducted by the Animal Ethics Committee of Suzhou Traditional Chinese Medicine Hospital.

#### 2.1.2. Molding

The water extracting method is used to extract the active ingredients of senna leaves: accurately weigh 2000 g of senna leaf pieces, soak in cold water for 3 times for 30 minutes, boil and simmer for 30 minutes, and then take 3000 ml through the filter. Sauté senna leaves and add 3000 ml of water for 30 minutes to filter the juice. The two liquids were concentrated and concentrated to 1 g of crude drug/ml of decoction 2000 ml and stored in a refrigerator at 4°C, which was ready for experimental use. Then, the 100% senna leaf ice water infusion was heated to 25°C in a water bath, and 36 mice in the three diarrhea groups were administered by intragastric administration in a volume of 10 ml/kg mouse twice a day for 10 days to develop a model of IBS-D [7].

#### 2.1.3. Grouping and Treatment

48 clean grade ICR male mice (20 ± 2 g) were randomly selected. After 3 days of adaptive feeding, mice were randomly divided into four groups (*n* = 12): control group, IBS-D group, compound of lactic acid bacteria group, and Jianpi mixture group. Before start the experiments, mice were observed and recorded daily for changes in feces characteristics, diet, body weight, and general conditions. After the confirmation of successful IBS-D model establishment, control group and IBS-D group were intragastrically administered with double-distilled water in a volume of 10 ml/kg. Compound of the lactic acid bacteria group was intragastrically administered with a compound of lactic acid bacteria (0.026 g/ml suspension) in a dose of 0.26 g/kg. Jianpi mixture group was intragastrically administered with the Jianpi mixture in a volume of 16 ml/kg. Mice were treated for 14 days. At the end of the treatment period, 5 mice were randomly selected from each group for 16S rDNA gene sequencing.

## 3. Experimental Medication

1. Jianpi mixture: composition. Jianpi mixture is prepared in Suzhou Traditional Chinese Medicine Hospital (Tangshen 5 g, Semen Pharbitidis 5 g, India bread 5 g, liquorice root 3 g, dried 5 g, common yam rhizome 5 g, 95% ethanol 24.88 ml, and potassium sorbate 0.084 g, sugar 4.2 g; batch number: 171205) and is authenticated by a pharmacist of traditional Chinese medicine in Suzhou Chinese Traditional Medicine Hospital. The composition of Jianpi mixture is shown in Table 1.

**Figure 1:**
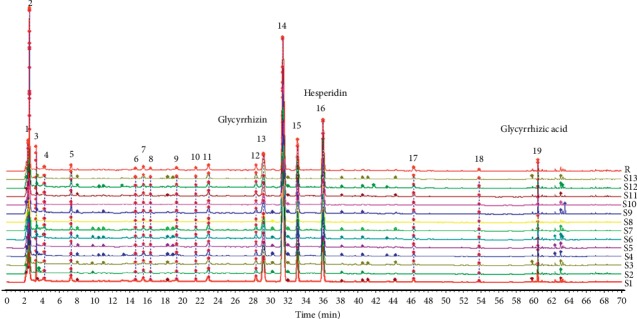
Fingerprint of Jianpi mixture. Each peak of 1–70 min represents the chromatographic peaks. According to reference, literature review, and identification, three of common peaks were glycyrrhizin (peak 13), hesperidin (peak 16), and glycyrrhizic acid (peak 19).

  The fingerprint of Jianpi mixture: the HPLC fingerprints of 13 batches Jianpi mixture samples (batches 190428 (S1), 180204 (S2), 180525 (S3), 180625 (S4), 180726 (S5), 180801 (S6), 180827 (S7), 180906 (S8), 181104 (S9), 181130 (S10), 190306 (S11), 190401 (S12), and 190427 (S13) provided by Suzhou Traditional Chinese Medicine Hospital) were introduced into the National Pharmacopoeia Committee “Chinese Medicine Chromatographic Fingerprint Similarity Evaluation System” (2012 edition) to establish the fingerprint of Jianpi mixture (Figure 1). Taking the sample (190428) as a reference map, after multipoint correction and data matching, the contrast fingerprint *R* was generated by the average method. The results show that the peak times of the main chromatographic peaks were basically the same. By comparing the chromatograms of each batch of samples, a total of 24 shared peaks were found. The fingerprint of Jianpi mixture is shown in Figure 1.

(2) Compound of lactic acid bacteria capsule: Jiangsu Meitong Pharmaceutical Co., Ltd., batch number: 180106.
(3) Sodium pentobarbital: supplied by Shanghai Chemical Reagent Purchasing and Supply Station Packing Factory (produced by Sigma company), batch number: 20089104.
(4) Senna leaf (General cargo, Guangdong): Suzhou Chunhuitang Pharmaceutical Co., Ltd., batch number: 170909.

## 4. General Observation and Analysis of Intestinal Microbiota

The general condition of each group of mice was observed, and the mental state, activity status, hair luster, and fecal nature of each group of mice were recorded regularly. The body weight, food intake, and Bristol score [8] (shown in Table 2) of each group of mice were recorded and analyzed.

### 4.1. Collection and Preparation of Feces and Mucosa in Mice

#### 4.1.1. Collection of Specimens

(1) Collection of feces: after the treatment, the mouse feces were taken, placed in a dry-frozen tube aseptically, and stored in the refrigerator at −80°C for preservation. (2) Collection of colonic mucosa: after the treatment, the mice were put to death under ether anesthesia, followed by laparotomy under aseptic conditions to extract the colon of the mice, rinse the colon feces, dissected and separated in the colon mucosa, and placed in the refrigerator at −80°C for preservation. (3) Preparation of samples: the feces before treatment in the control group, IBS-D group, compound of lactic acid bacteria group, and Jianpi mixture group were, respectively, labeled as A1, A2, A3, and A4. After treatment, feces were labeled as B1, B2, B3, and B4. Colonic mucosa was labeled as C1, C2, C3, and C4 after treatment.

#### 4.1.2. Preparation of Intestinal Microbiota Analysis Specimens

Take the refrigerated samples and reconstitute at room temperature. Sampling the weight, the proportion of homogenate is 10% (i.e., 1 g feces plus 9 ml homogenate); the homogenate was selected from PBS (pH = 7.2–7.4, concentration: 0.01 mol/L) using a tissue homogenizer, homogenizing on an ice bath, centrifuging at 5000 rpm for 15 min, and the supernatant is taken for further analysis.

## 5. Laboratory Apparatus

352 type microplate reader: Finland (Labsystems Multiskan MS); AC8 plate washer: Finland (Thermo Labsystems); TG16 W centrifuge: microhigh speed centrifuge (Domestic); GNP-9080 type incubator: water-proof constant temperature incubator (Domestic); *Z* 320K low-temperature high-speed centrifuge: Germany HERMLE company production; Sigma 1–13 microcentrifuge: American Sigma company production; electrooptical analysis balance (BD-211D), domestic; AX-26DR one thousandth analysis balance, Switzerland, produced by Mettler Toledo; ABI-7300 real-time detector, produced by ABI; TG-16M cryogenic refrigerated centrifuge, produced by Shanghai Luxiangyi Centrifuge Instrument Co., Ltd.; P2, P10, P20, P100, P200, and P1000 Liquid gun, Gilson P-type pipette company production; K30 vortex oscillator: Qingpu West Instrument Factory; PRO200 electric homogenizer: FLUKO company production; precision electronic balance: Zhuo Jing, Shanghai; oscillator vortex-5: Its Linbell, Haimen; electronic constant temperature stainless steel water bath: HHS-2S, Shanghai; Eppendorf centrifuge: Eppendorf, Germany; electrophoresis and gel imager: Bio Rad, USA; ABI9700 ladder PCR instrument: ABI, USA; Axygen Gel Recovery Kit: Axygen, USA; FTC-3000 TM real-time PCR: Maple Ridge, Shanghai; MiSeq Sequencer: Illumina, USA.

## 6. Analysis Method

First, genomic DNA extraction was performed on all samples using the QIAamp DNA Stool Mini Kit, followed by bidirectional sequencing according to the Illumina MiSeq high-throughput sequencing requirement, and two-step PCR amplification was used: (1) specific primers (F inner primer: 5′-TTCCCTACACGACGCTCTTCCGATCT-specific primer-3′, *R* inner primer: 5′-GAGTTCCTTGGCACCCGAGAATTCCA-specific primer-3′) are used to amplify the target fragment, and the target fragment was subjected to gel recovery. (2) The recovered product was used as a template for secondary PCR amplification (F lateral primer: 5′-AATGATACGGCGACCACCGAGATCTACAC-barcode-TCTTTCCCTACACGACGCTC-3′, *R* lateral primer: 5′-CAAGCAGAAGACGGCATACGAGAT-barcode-GTGACTGGAGTTCCTTGGCACCCGAGA-3′). The aim is to add the linker, sequencing primer, and barcode required for the Illumina platform sequencing to both ends of the target fragment. All PCR products were recovered using the AxyPrepDNA Gel Recovery Kit and quantified by FTC-3000TM real-time PCR instrument, and the samples were prepared by mixing the specimens in equimolar ratios. Second, Illumina MiSeq 2 × 300 bp high-throughput sequencing and bioinformatics analysis were performed.

## 7. Statistics and Processing of Data

R 3.4.1 Language mapping software is used to analyze intestinal microbiota; SPSS 22 statistical analysis is performed, and the data were plotted in the figure as mean ± standard error (SE). Wilcox and *T* test were used to analyze the differences between the groups. A *P* value <0.05 was considered to be statistically significant.

## 8. Results and Discussion

### 8.1. Results

#### 8.1.1. Record of General Condition, Weight, Food Intake, and Bristol Score

*(1) General Condition of Each Group of Mice*. The feces characteristics and food intake of the mice were monitored and recorded every day. It can be seen that diarrhea in mice in compound of the lactic acid bacteria group and in Jianpi mixture group had been improved after treatments. The feces of the IBS-D group were still sparse and not formed, and the hair was gray and temperate. The hair and behavioral status of the compound of lactic acid bacteria group and Jianpi mixture group were improved compared with the IBS-D group. The feces of the compound lactic acid bacteria group began to be normal after 7 days, and the feces of the Jianpi mixture group are formed, but the water was still more.

*(2) Body Weight Changes in Each Group of Mice*. Body weight before and after administration can be seen in Table 3. After modeling, the body weight of mice in each group was significantly lower than that of the control group (*P* < 0.05). The above information initially confirmed the success of the IBS-D model. After administration, the body weight of compound of the lactic acid bacteria group and Jianpi mixture group was higher than that of IBS-D group on the 7th and 14th days after treatments (*P* < 0.05).

*(3) Changes in Food Intake of Each Group of Mice*. As can be seen from Table 4, after 10 days of modeling, the increase in food intake in the IBS-D group was significantly different from that in the control group (*P* < 0.05). After 7th and 14th days of treatment, the intake of Jianpi mixture group increased compared with the IBS-D group (*P* < 0.05).

*(4) Changes in Bristol Scores of Each Group of Mice.* It can be seen from Table 5 that, after 10 days of modeling, the Bristol scores of each group were significantly increased (*P* < 0.05). After 14 days of treatment, the lactic acid bacteria group and the Jianpi mixture group were significantly lower than the IBS-D group (*P* < 0.05).

#### 8.1.2. Sample DNA Quality Control and PCR Amplification

**Figure 2:**
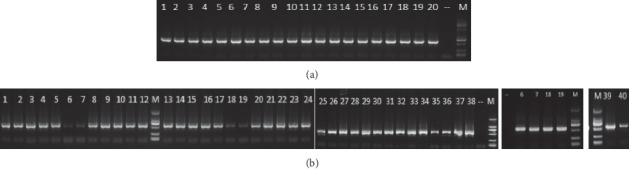
Electrophoretogram of PCR amplification of bacterial DNA in 60 samples. (a) Electrophoretogram of secondary PCR amplification of fecal bacterial DNA in 20 samples before treatment. Each band of 1∼20 represents the molecular weight of fecal bacterial DNA in the sample of mice before treatment. Marker is DL2000. The top to bottom band is 2000 bp, 1000 bp, 750 bp, 500 bp, 250 bp, and 100 bp; the loading is 3 *µ*L, and the bright band is 30 ng/*µ*L; the remaining strips are all 10 ng/*µ*L. (b) Electrophoretogram of secondary PCR amplification of bacterial DNA in 40 samples after treatment. Each band of 1∼20 represents the molecular weight of bacterial DNA in the fecal sample of the mice after treatment, each band of 21–40 represents the molecular weight of bacterial DNA of the colonic mucosa of the mice after treatment, and marker is DL2000. The top to bottom band is 2000 bp, 1000 bp, 750 bp, 500 bp, 250 bp, and 100 bp; the loading is 3 *µ*L, the bright band is 30 ng/*µ*L, and the remaining bands are 10 ng/*µ*L.

Electrophoretogram of PCR amplification of bacterial DNA is shown in Figures 2(a) and, 2(b). After the second PCR amplification, the bands were single, the brightness was moderate, and the molecular weight of each band was between 750 and 500 bp, which was in line with the expected results, and the subsequent gel recovery experiments were carried out.

#### 8.1.3. Analysis of DNA Sequencing Sequence Results of All Mouse Samples

**Figure 3:**
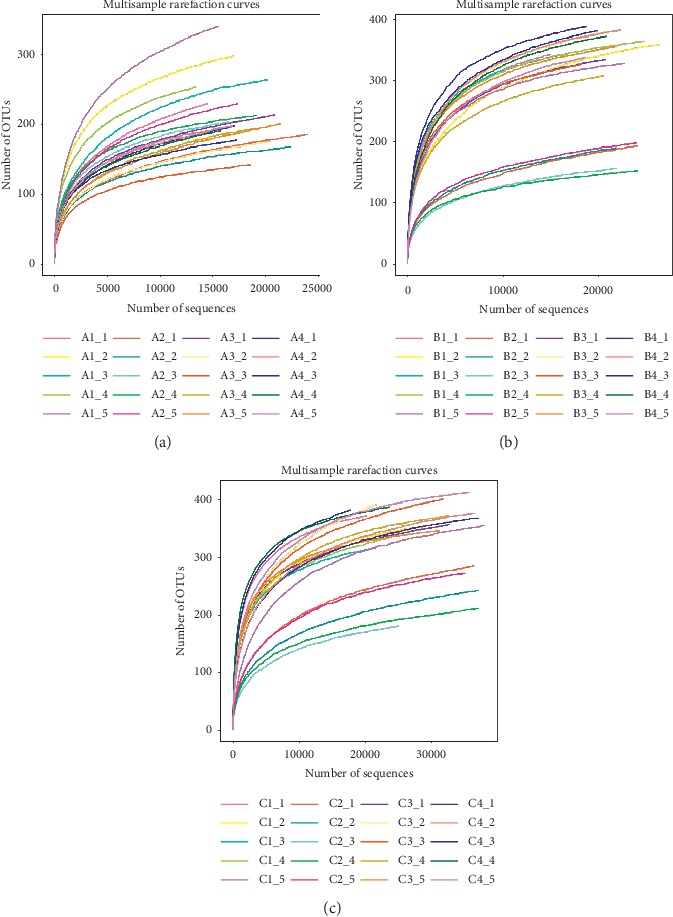
Analysis of intestinal microbiota diversity in mice: (a) rarefaction curve of group A (*n* = 5); (b) rarefaction curve of group B (*n* = 5); (c) rarefaction curve of group C (*n* = 5). The abscissa represents the amount of random sampling, and the ordinate represents the OTU number.

*(1) Analysis of Intestinal Microbiota Diversity of Mice in Groups A, B, and C*. The sequencing is a statistical analysis of bioinformatics for ≥97% of OTU. To verify that the amount of sequencing data is sufficient to reflect sample species diversity, the mothur software is used to map the rarefaction curve (see Figures 3(a)–3(c)).

The rarefaction curve is used to evaluate whether the amount of sequencing is reasonable. The flatter the curve, the more adequate the sequencing depth, and vice versa, indicating that there are still more species not detected by sequencing. It can be seen from Figures 3(a)–3(c) that all the sequencing amounts have obvious turning points around 1000 and then enter the plateau stage, indicating that the sequencing has become saturated, and increasing the sequencing data can no longer find more OTUs. The sequencing depth is sufficient, and the samples all have good species richness.

**Figure 4:**
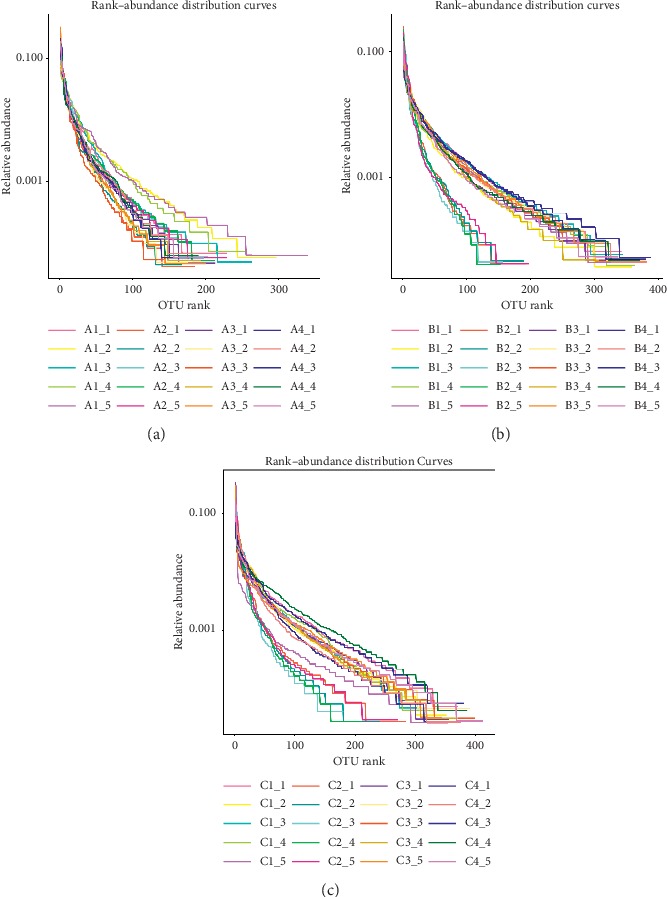
Analysis of intestinal microbiota diversity in mice: (a) rank-abundance of group A (*n* = 5); (b) rank-abundance of group B (*n* = 5); (c) rank-abundance of group C (*n* = 5). The abscissa represents the OTU rank, and the ordinate represents the relative abundance.

In order to verify the diversity of the bacteria in this sample to meet the requirements of the experiment, the rank-abundance curve was drawn (see Figures 4(a)–4(c)). The rank-abundance curve mainly reflects two aspects of species diversity, namely, the species richness and species evenness contained in the sample. The wider the curve, the higher the species richness; the flatter the curve, the higher the species evenness. It can be seen from Figures 4(a)–4(c) that the curves of most of the samples were wider in the horizontal direction, and the downward trend is more gradual, indicating that species richness and species evenness of the bacteria in the 60 samples were reasonable.

**Figure 5:**
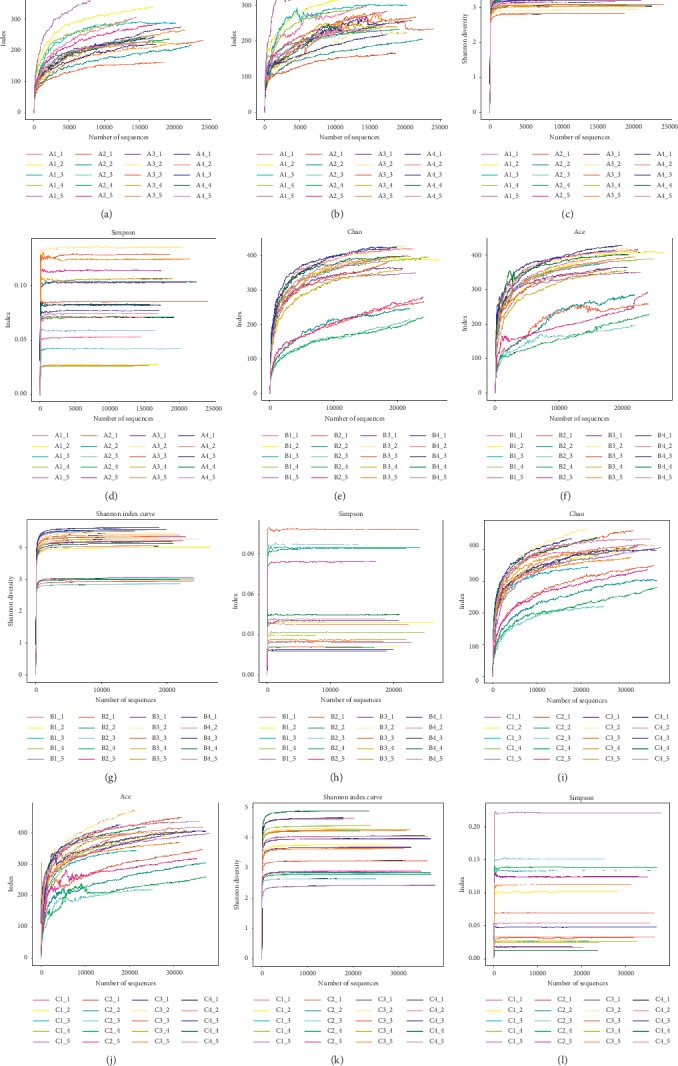
Index of alpha diversity analysis of intestinal microbiota in groups A, B, and C. (a) The Chao index curve of group A (*n* = 5); (b) the Ace index curve of group A (*n* = 5); (c) the Shannon index curve of group A (*n* = 5); (d) the Simpson index curve of group A (*n* = 5). (e) The Chao index curve of group B (*n* = 5); (f) the Ace index curve of group B (*n* = 5); (g) the Shannon index curve of group B (*n* = 5); (h) the Simpson index curve of group B (*n* = 5). (i) The Chao index curve of group C (*n* = 5); (j) the Ace index curve of group C (*n* = 5); (k) the Shannon index curve of group C (*n* = 5); (l) the Simpson index curve of group C (*n* = 5).

*(2) Comparison of Intestinal Microbiota of Mice in Groups A, B, and C*. Biodiversity mainly uses alpha diversity analysis based on OTU clustering results. Alpha diversity includes Chao index, Ace index, Shannon index, and Simpson index (see Figures 5(a)–5(l)). Among them, the Chao index and the Ace index mainly explain the species richness of the intestinal microbiota in samples, while the Shannon index and the Simpson index explain the species diversity of the intestinal microbiota. The comparison between Chao index, Ace index, Shannon index, and Simpson index shows that the biological species richness and diversity of these samples are high (Figures 5(a)–5(l)).

**Figure 6:**
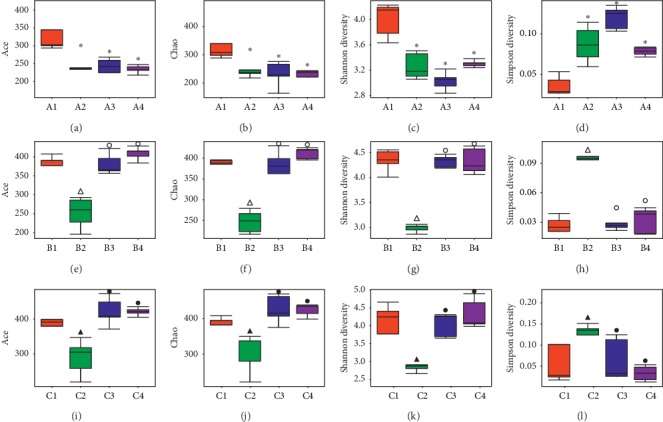
Box plots of the diversity of alpha diversity analysis of intestinal microbiota in groups A, B, and C. (a) The Ace index box plot of group A (*n* = 5); (b) the Chao index box plot of group A (*n* = 5); (c) the Shannon index box plot of group A (*n* = 5); (d) the Simpson index box plot of group A (*n* = 5). (e) The Ace index box plot of group A (*n* = 5); (f) the Chao index box plot of group A (*n* = 5); (g) the Shannon index box plot of group A (*n* = 5); (h) the Simpson index box plot of group A (*n* = 5). (i) The Ace index box plot of group A (*n* = 5); (j) the Chao index box plot of group A (*n* = 5); (k) the Shannon index box plot of group A (*n* = 5); (l) the Simpson index box plot of group A (*n* = 5). ^*∗*^*P* < 0.05, compared to the A1 group. ^Δ^*P* < 0.05, compared with the B1 group. °*P* < 0.05, compared with the B2 group. ^▲^*P* < 0.05, compared with the C1 group. ^•^*P* < 0.05, compared to the C2 group.

According to the differences between groups of mice, box plots were drawn (see Figures 6(a)–6(l). It can be seen that the species richness and species diversity of A2, A3, and A4 were lower than those of A1 (*P* < 0.05); compared with B1, the species richness and diversity of the B2 group were lower (*P* < 0.05). Compared with B2, the richness and diversity of B3 and B4 groups were higher (*P* < 0.05). Compared with C1, the species richness and diversity of C2 decreased (*P* < 0.05); compared with C2, the species richness and diversity of C3 and C4 groups increased (*P* < 0.05).

*(3) Species Information and Difference Analysis of Intestinal Microbiota of Mice in Groups of A, B, and C*. According to the results of taxonomic analysis of species information, it can be known that one or several samples are compared at different biological classification levels. Statistical methods can be used to observe and analyze the community structure of the sample at the six different taxonomic levels of the phylum, class, order, family, genus, and species.

**Figure 7:**
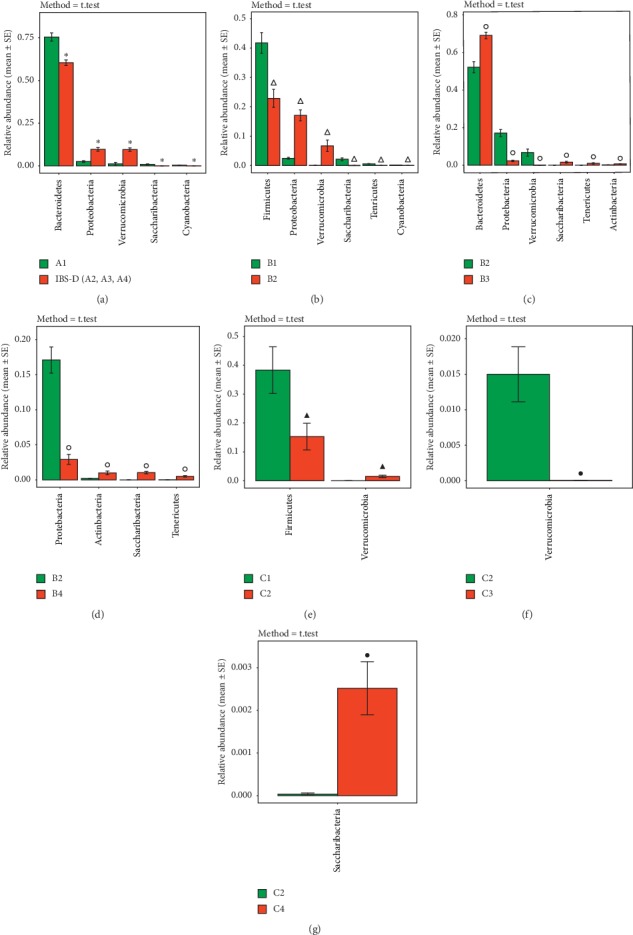
Distribution and difference of intestinal microbiota of mice in groups A, B, and C at the phylum level. (a) histogram of the difference analysis of group A at the phylum level (*n* = 5). (b) A histogram of the difference analysis of groups B1 and B2 at the phylum level (*n* = 5); (c) a histogram of the difference analysis of groups B2 and B3 at the phylum level (*n* = 5); (d) a histogram of the difference analysis of groups B2 and B4 at the phylum level (*n* = 5). (e) A histogram of the difference analysis of groups C1 and C2 at the phylum level (*n* = 5); (f) a histogram of the difference analysis of groups C2 and C3 at the phylum level (*n* = 5); (g) a histogram of the difference analysis of groups C2 and C4 at the phylum level (*n* = 5). ^*∗*^*P* < 0.05, compared to the A1 group. ^Δ^*P* < 0.05, compared with the B1 group. °*P* < 0.05, compared with the B2 group. ^▲^*P* < 0.05, compared with the C1 group. ^•^*P* < 0.05, compared to the C2 group.

*(4) Distribution and Difference of Intestinal Microbiota of Mice in Groups A, B, and C at the Phylum Level*. According to the test results, except for the unclassified bacteria, the first nine phylum with the highest relative abundance of mice after treatment are Bacteroidetes, Firmicutes, Proteobacteria, Verrucomicrobia, Saccharibacteria, Deferribacteres, Actinobacteria, Tenericutes, and Cyanobacteria. After species difference analysis, select the species with *P* < 0.05 to draw the histogram (see Figures 7(a)–7(g)). It can be seen that, compared with the control group, the abundance of Proteobacteria and Verrucomicrobia in the fecal of IBS-D mice (A2, A3, and A4) was upregulated (*P* < 0.05) and the abundance of Bacteroidetes, Saccharibacteria, and Cyanobacteria was downregulated (*P* < 0.05). Comparing B1 with B2, the abundance of Proteobacteria and Verrucomicrobia in the B2 group was upregulated (*P* < 0.05) and the abundance of Firmicutes, Saccharibacteria, Tenericutes, and Cyanobacteria in the B2 group was downregulated (*P* < 0.05). Comparing B2 with B3, the abundance of Bacteroidetes, Saccharibacteria, Tenericutes, and Actinobacteria in the B3 group was upregulated (*P* < 0.05) and the abundance of Proteobacteria and Verrucomicrobia in the B3 group was downregulated (*P* < 0.05). Comparing B2 with B4, the abundance of Tenericutes, Actinobacteria, and Saccharibacteria in the B4 group was upregulated (*P* < 0.05) and the abundance of Proteobacteria in the B4 group was downregulated (*P* < 0.05). Compared with C1, the abundance of Verrucomicrobia in the C2 group was upregulated (*P* < 0.05) and the abundance of Firmicutes in the C2 group was downregulated (*P* < 0.05). Compared with C2, the abundance of Verrucomicrobia in the C3 group was downregulated (*P* < 0.05) and the abundance of Saccharibacteria in the C4 group was upregulated (*P* < 0.05).

**Figure 8:**
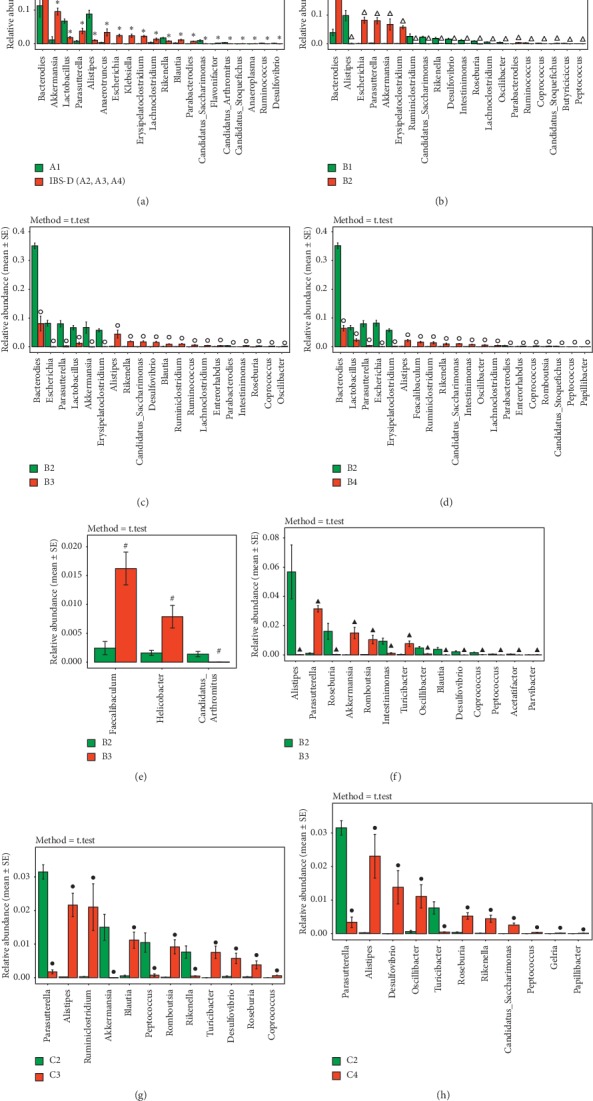
Distribution and difference of intestinal microbiota of mice in groups A, B, and C at the genus level. (a) histogram of the difference analysis of group A at the genus level (*n* = 5). (b) A histogram of the difference analysis of groups B1 and B2 at the genus level (*n* = 5); (c) a histogram of the difference analysis of groups B2 and B3 at the genus level (*n* = 5); (d) a histogram of the difference analysis of groups B2 and B4 at the genus level (*n* = 5); (e) a histogram of the difference analysis of groups B3 and B4 at the genus level (*n* = 5). (f) A histogram of the difference analysis of groups C1 and C2 at the genus level (*n* = 5); (g) a histogram of the difference analysis of groups C2 and C3 at the genus level (*n* = 5); (h) a histogram of the difference analysis of groups C2 and C4 at the genus level (*n* = 5). ^*∗*^*P* < 0.05, compared to the A1 group. ^Δ^*P* < 0.05, compared with the B1 group. °*P* < 0.05, compared with the B2 group. ^#^*P* < 0.05, compared with the B3 group. ^▲^*P* < 0.05, compared with the C1 group. ^•^*P* < 0.05, compared to the C2 group.

*(5) Distribution and Difference of Intestinal Microbiota of Mice in Groups A, B, and C at the Genus Level*. At the genus level, it was found that a total of 126 species of genus were obtained from the intestinal fecal microbiota of mice, and 69 species of genus were obtained from the colonic mucosa colonization of mice. Among the higher abundances are unclassified Bacteroides, Helicobacter, Akkermansia, Alistipes, Lactobacillus, Escherichia, Anaerotruncus, Parasutterella, Mucispirillum, and Erysipelatoclostridium. Through significant difference analysis between groups (MetaStats), select the species with *P* < 0.05 for the histogram mapping (select the bacteria with the top 20 abundances) (see Figures 8(a)–8(h)). Compared with the control group, Bacteroides, Akkermansia, Parasutterella, Anaerotruncus, Escherichia, Klebsiella, Erysipelatoclostridium, Lachnoclostridium, Blautia, Parabacteroides, Flavonifractor, Candidatus Stoquefichus, and Anaeroplasma in the fecal of IBS-D mice with (A2, A3, and A4) before treatment was upregulated (*P* < 0.05) and the abundance of Lactobacillus, Alistipes, Rikenella, Candidatus Saccharimonas, Candidatus Arthromitus, Ruminococcus, and Desulfovibrio was downregulated (*P* < 0.05). Comparing B1 with B2, the abundance of Bacteroides, Parasutterella, Escherichia, Erysipelatoclostridium, Akkermansia, Parabacteroides, Candidatus Stoquefichus, and Anaerofustis of the B2 group was upregulated (*P* < 0.05) and the abundance of Alistipes, Ruminiclostridium, Candidatus Saccharimonas, Rikenella, Desulfovibrio, Intestinimonas, Roseburia, Lachnoclostridium, Oscillibacter, Ruminococcus, Coprococcus, and Peptococcus of the B2 group was downregulated (*P* < 0.05). Comparing B2 with B3, the abundance of Alistipes, Ruminiclostridium, Candidatus Saccharimonas, Rikenella, Desulfovibrio, Intestinimonas, Roseburia, Lachnoclostridium, Oscillibacter, Blautia, Ruminococcus, Coprococcus, Enterorhabdus, Parvibacter, Peptococcus, Streptococcus, and Papillibacter of the B3 group was upregulated (*P* < 0.05) and the abundance of Lactobacillus, Bacteroides, Parasutterella, Escherichia, Erysipelatoclostridium, Akkermansia, Caproiciproducens, Parabacteroides, and Candidatus Stoquefichus of the B3 group was downregulated (*P* < 0.05). Comparing B2 with B4, the abundance of Alistipes, Ruminiclostridium, Candidatus Saccharimonas, Rikenella, Intestinimonas, Lachnoclostridium, Oscillibacter, Coprococcus, Faecalibaculum, Enterorhabdus, Peptococcus, Romboutsia, and Papillibacter of the B4 group was upregulated (*P* < 0.05) and the abundance of Lactobacillus, Bacteroides, Parasutterella, Escherichia, Erysipelatoclostridium, Parabacteroides, and Candidatus Stoquefichus of the B4 group was downregulated (*P* < 0.05). Comparing B3 with B4, the abundance of Helicobacter and Faecalibaculum of the B4 group was upregulated (*P* < 0.05) and the abundance of Candidatus Arthromitus of the B4 group was downregulated (*P* < 0.05). Comparing C1 with C2, the abundance of Parabacteroides, Akkermansia, Romboutsia, Turicibacter, and Patescibacteria of the C2 group was upregulated (*P* < 0.05) and the abundance of Alistipes, Roseburia, Intestinimonas, Oscillibacter, Blautia, Desulfovibrio, Coprococcus, Peptococcus, and Acetatifactor of the C2 group was downregulated (*P* < 0.05). Compared with C2, the abundance of Alistipes, Ruminiclostridium, Blautia, Rikenella, Desulfovibrio, Roseburia, Coprococcus, and Peptococcus of the C3 group was upregulated (*P* < 0.05); the abundance of Parasutterella, Akkermansia, Romboutsia, and Turicibacter of the C3 group was downregulated (*P* < 0.05); the abundance of Alistipes, Desulfovibrio, Oscillibacter, Roseburia, Rikenella, Candidatus Saccharimonas, Peptococcus, Gelria, and Paludibacter of the C4 group was upregulated (*P* < 0.05); and the abundance of Parasutterella and Turicibacter of the C4 group was downregulated (*P* < 0.05).

**Figure 9:**
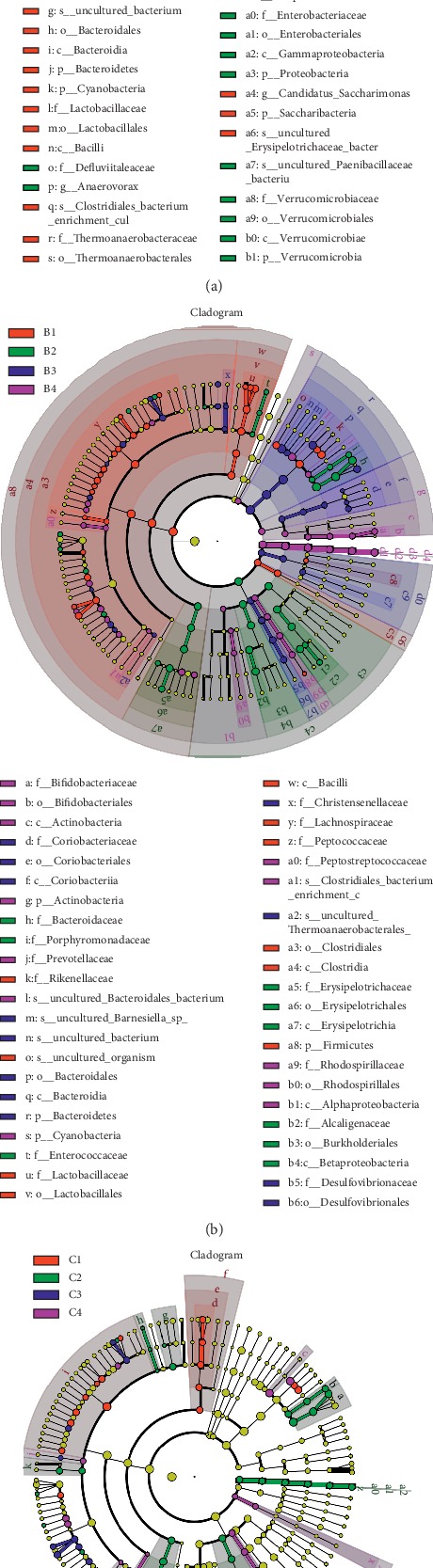
Difference analysis of intestinal microbiota structure of mice in groups A, B, and C: (a) LEfSE tree diagram of group A (*n* = 5); (b) LEfSE tree diagram of group B (*n* = 5); (c) LEfSE tree diagram of group C (*n* = 5). This circle diagram of radiation from inside to outside is a clustering tree, which in turn represents the classification level of phylum, class, order, family, genus, and species. Each small circle on a different circle layer represents a classification at that level, and the diameter of the small circle is proportional to the relative abundance of the classification. In Figure 9 (a), the red area indicates the group A1 and the green area indicates the IBS-D group (A2, A3, and A4); in Figures 9 (b) and (c), the different red areas in the tree diagram represent the BI and C1 groups, the green represents the B2 and C2 groups, the blue area represents the B3 and C3 groups, and the purple area represents the B4 and C4 groups. The yellow represents the species with no differences between the components. The red node represents the dominant microbiota in the red group, and the green node represents the dominant microbiota in the green group. Each node is identified by a lowercase letter, and a species annotation is made on the right side of the tree.

*(6) Difference Analysis of Intestinal Microbiota Structure of Mice in Groups A, B, and C*. In order to further analyze the composition of the intestinal microbiota between each group and the distribution difference of each species and to identify the dominant microbiota of each group, the difference analysis between the groups was adopted (LDA effect size). The analysis method can achieve comparison between multiple groups and can also perform subgroup comparison analysis within the group, so as to find the genus that plays a major role in each group of samples and species with significant differences in abundance between groups (i.e., biomarker). LEfSE (LDA effect size) tree drawing is done using LEfSE software. It can be found in Figures 9(a)–9(c) that the main role of the A1 group and differences from other groups are Prevotellaceae, Rikenellaceae, uncultured Bacteroidales bacterium, uncultured Barnesiella sp., uncultured bacterium, Bacteroidales, Bacteroidia, Bacteroidetes, Cyanobacteria, Lactobacillaceae, Lactobacillales, Bacilli, Clostridiales-bacterium-enrichment-culture-clone-06-1235251–76, Thermoanaerobacteraceae, Thermoanaerobacterales, Alphaproteobacteria, Candidatus Saccharimonas, Saccharibacteria, and uncultured Erysipelotrichaceae bacterium. The main role of the IBS-D group and differences from other groups are Bacteroidaceae, Porphyromonadaceae, Defluviitaleaceae, Anaerovorax, Erysipelotrichaceae, Erysipelotrichales, Erysipelotrichia, Alcaligenaceae, Burkholderiales, Betaproteobacteria, Enterobacteriaceae, Enterobacteriales, Gammaproteobacteria, Proteobacteria, uncultured Paenibacillaceae bacterium, Verrucomicrobiaceae, Verrucomicrobiales, Verrucomicrobiae, and Verrucomicrobia. The main role of the B1 group and differences from other groups are Rikenellaceae, uncultured organism, Lactobacillaceae, Lactobacillales, Bacilli, Lachnospiraceae, Peptococcaceae, Clostridiales, Clostridia, and Firmicutes. The main role of the B2 group and differences from other groups are Bacteroidaceae, Porphyromonadaceae, Enterococcaceae, Erysipelotrichaceae, Erysipelotrichales, Erysipelotrichia, Alcaligenaceae, Burkholderiales, and Betaproteobacteria. The main role of the B3 group and differences from other groups are Coriobacteriaceae, Coriobacteriales, Coriobacteriia, Bacteroidales, Bacteroidetes, Christensenellaceae, Desulfovibrionales, and Desulfovibrionaceae. The main role of the B4 group and differences from other groups are Bifidobacteriaceae, Bifidobacteriales, Actinobacteria, Prevotellaceae, Cyanobacteria, Peptostreptococcaceae, Clostridiales, Rhodospirillaceae, Rhodospirillales, and Alphaproteobacteria. The main role of the C1 group and differences from other groups are Lactobacillaceae, Lactobacillales, Bacilli, and Lachnospiraceae. The main role of the C2 group and differences from other groups are Bacteroidaceae, Porphyromonadaceae, Clostridiaceae, Eubacteriaceae, Peptostreptococcaceae, Erysipelotrichaceae, Erysipelotrichales, Erysipelotrichia, Alcaligenaceae, Burkholderiales, Betaproteobacteria, Verrucomicrobiaceae, Verrucomicrobiales, Verrucomicrobiae, and Verrucomicrobia. The main role of the C4 group and differences from other groups are uncultured_Bacteroidales_bacterium, Peptococcaceae, Anaerovorax, Thermoanaerobacteraceae, Thermoanaerobacterales, Desulfovibrionaceae, Desulfovibrionales, Deltaproteobacteria, Candidatus Saccharimonas, and Saccharibacteria.

### 8.2. Discussion

In the present study, our results demonstrated that IBS-D mice showed a lower intestinal microbiota diversity compared with healthy mice. Treatment with Jianpi mixture alleviates the symptoms of IBS-D, accompanied with restoration of diversity of intestinal microbiota and regulation of proportion of dominant microbiota close to health condition. These results indicate that intestinal microbiota abnormality may be involved in the pathogenesis of IBS-D.

80% of the symbiotic microorganisms were distributed on and in the body alive in the digestive tract. The species is more than 1000 species, weighing up to 2 kg, and the total number of cells is more than 10^14^, which is about 1.3 times the number of human cells [9]. The number of these microbes and the total number of genes are so large that they are destined to affect human health. The related mechanisms of intestinal microecology involved in the pathogenesis of IBS-D mainly include changing mucosal permeability, activating intestinal mucosal immune response, affecting brain-intestinal axis, and changing gastrointestinal motility. Carroll et al. investigated microorganisms in the fecal and colonic mucosa of patients with IBS-D and reported that their microbial community was significantly different from the healthy group, and the microbial diversity in fecal samples was significantly reduced in the IBS-D group [10]. Our observation also demonstrated that the abundance and diversity of intestinal microbiota of IBS-D mice were lower than healthy mice, which is consistent with the existing literatures.

Because the pathogenesis of IBS-D is not completely clear, clinical treatments are mainly based on symptom such as antispasmodic agents, antidiarrheal drugs, intestinal motility sensory agents, and psychotropic drugs, but the efficacy is uncertain. Microecological modulators have been used clinically to intervene the intestinal microbiota for the treatment of some gastrointestinal diseases. Microecological modulators include probiotics, prebiotics, and symbiotics, mainly by increasing the number of beneficial bacteria in the host gut to regulate ecological disorders and maintain microecological balance [11]. Yao et al. [12] postulated that probiotics combined with gastrointestinal motility drugs can improve the curative effect, especially in improving abdominal pain and diarrhea symptoms, but the improvement of abdominal distension symptoms is not obvious. The use of probiotics alone has a certain effect on IBS. Other studies have shown that Si Ni San combined with Tong Xie Yao Fang in treating diarrhea-type irritable bowel syndrome can improve patient symptoms with abdominal pain, defecation urgency, and other symptoms and also has a positive effect on Escherichia coli, Lactobacillus, and Bifidobacteria in the intestinal microbiota [13].

With the in-depth study of contemporary Chinese medicine workers, the essential relationship between TCM and microecology is gradually being revealed [14]. In recent years, the regulation of intestinal microecology by the spleen-strengthening formula of TCM has received increasing attention.

Jianpi mixture is developed by a doctor of TCM named Huang Yifeng. The main drugs are Tangshen, Semen Pharbitidis, India bread, liquorice root, dried, and common yam rhizome. Studies have found that Tangshen pilosula polysaccharide and Tangshen saponins can promote the growth of probiotics in the intestine, inhibit the colonization of pathogenic bacteria, downregulate the F/B ratio, and restore the intestinal homeostasis [15]. Semen Pharbitidis has the effect of strengthening the spleen and qi. Ye et al. [16] and other researchers found that Semen Pharbitidis can significantly promote the proliferation of Lactobacillus acidophilus and inhibit the proliferation of Escherichia coli. India bread has the effect of dehumidification and is good for the spleen and heart. Pharmacological studies have found that India bread has an inhibitory effect on common Escherichia coli, Staphylococcus aureus, and Pseudomonas aeruginosa [17]. Liquorice root has the effect of replenishing vital energy, relieving pain, and reconciling various medicines. Studies have found that high-dose licorice aqueous extract can downregulate the proportion of Bacteroides, Proteobacteria, and Desulfovibrio and increase the proportion of Firmicutes [18]. Researches shows that dried has a certain inhibitory effect on the spontaneous activity of the gastrointestinal tract and can obviously antagonize contraction and spasm of the intestine caused by histamine or acetylcholine and also has certain antiallergic effects. These effects are one of the pharmacological foundations of dried to regulate qi and spleen, eliminating phlegm [19]. Common yam rhizome has the effect of strengthening the spleen and qi, nourishing the stomach, and healing diarrhea. Common yam rhizome polysaccharide can regulate intestinal microbiota; long-term gavage of common yam rhizome polysaccharide can inhibit the number of bacteria of Enterobacteria and Enterococcus and promote the proliferation of probiotics Bifidobacterium and Lactobacillus [20]. In summary, Jianpi mixture has the effect of strengthening spleen and qi, healing diarrhea, and its mechanism may be related to its role in regulating intestinal microecology. In the present study, we clearly demonstrated that Jianpi mixture could alleviate symptoms of IBS-D in the mouse model. In addition, Jianpi mixture can partially normalized the diversity of intestinal microbiota at various levels, which has been reported to be highly related to occurrence of IBS-D [6, 21–31].

Our present study basically clarified that the IBS-D mice has significantly shifted intestinal microbiota compared with healthy mice, showing a decline in a large number of beneficial bacteria and an increase in a large number of harmful bacteria. After the intervention of Jianpi mixture, the intestinal microbiota diversity of IBS-D mice was restored, and the proportion of each dominant microbiota was regulated to close health state. Our present study provides a novel insight into the pathogenesis of IBS-D and provides laboratory evidence for clinical application Jianpi mixture.

## Figures and Tables

**Table 1:** Composition of Jianpi mixture.

Chinese name	Latin name	English name	Amount (g)	Place of origin
Dang Shen	*Radix codonopsis pilosula*	Tangshen	5	Gansu, China
Bai Zhu	*Rhizoma Atractylodis macrocephalae*	Semen pharbitidis	5	Zhejiang, China
Fu Ling	*Sclerotium poriae cocos*	India bread	5	Anhui, China
Zhi Gan Cao	*Radix glycyrrhizae preparata*	Liquorice root	3	Inner Mongolia, China
Chen Pi	*Percarpium citri reticulatae*	Dried common	5	Jiangsu, China
Shan Yao	*Radix dioscoreae oppositae*	Yam rhizome	5	Henan, China

**Table 2:** Bristol classification integration standards.

State	Type	Description	Integration
Constipation	Type 1	Nut-shaped, scattered hard blocks	1
	Type 2	Block shape	2
	Type 3	Sausage-shaped with cracks in the surface	3
Normal	Type 4	Sausage-shaped, smooth, and soft	4
	Type 5	A soft lump with clear edges	5
Diarrhea	Type 6	Pasty or muddy, with blurred edges	6
	Type 7	Watery with no solids	7

**Table 3:** Body weight changes in each group of mice (*n*=12^−^*x* ± *s*).

Group	Phase point weight (g)		
	Day 1 of administration	Day 7 of administration	Day 14 of administration
Control group	26.0 ± 1.6	31.6 ± 2.3	37.3 ± 1.4
IBS-D group	23.8 ± 1.1^*∗*^	27.0 ± 1.8^*∗*^	33. 3 ± 1.4^*∗*^
Compound of lactic acid bacteria group	23.4 ± 1.0^*∗*^	28.3 ± 1.2^*∗*^	34.4 ± 1.4^*∗*^
Jianpi mixture group	24.3 ± 1.2^*∗*^	29.4 ± 1.8^*∗*^^#^	34.9 ± 2.1^*∗*^^#^

^*∗*^ *P* < 0.05 compared with the control group on day 1, day 7, and day 14. ^#^*P* < 0.05 compared with the IBS-D group on day 7 and day 14.

**Table 4:** Food intake changes in each group of mice (*n*=12^−^*x* ± *s*).

Group	Food intake (g)		
	Day 1 of administration	Day 7 of administration	Day 14 of administration
Control group	2.18 ± 0.14	2.58 ± 0.15	3.03 ± 0.17
IBS-D group	1.77 ± 0.14^*∗*^	2.10 ± 0.21^*∗*^	2.42 ± 0.18^*∗*^
Compound of lactic acid bacteria group	1.68 ± 0.09^*∗*^	2.24 ± 0.19^*∗*^	2.60 ± 0.26^*∗*^
Jianpi mixture group	1.68 ± 0.17^*∗*^	2.28 ± 0.20^*∗*^^#^	2.80 ± 0.32^*∗*^^#^

^*∗*^ *P* < 0.05 compared with the control group on day 1, day 7, and day 14. ^#^*P* < 0.05 compared with the IBS-D group on day 7 and day 14.

**Table 5:** Bristol scores changes in each group of mice (*n*=12^−^*x* ± *s*).

Group	Bristol scores		
	Day 1 of administration	Day 7 of administration	Day 14 of administration
Control group	3.8 ± 0.6	3.7 ± 0.7	3.8 ± 0.7
IBS-D group	6.8 ± 0.5^*∗*^	6.6 ± 0.7^*∗*^	6.6 ± 0.5^*∗*^
Compound of lactic acid bacteria group	6.7 ± 0.5^*∗*^	5.4 ± 0.7^*∗*^^#^	4.5 ± 0.5^*∗*^^#^
Jianpi mixture group	6.7 ± 0.5^*∗*^	5.3 ± 0.7^*∗*^^#^	4.2 ± 0.4^#^

^*∗*^ *P* < 0.05 compared with the control group on day 1, day 7, and day 14. ^#^*P* < 0.05 compared with the IBS-D group on day 7 and day 14.

## Data Availability

The data used to support the findings of this study are included within the article.
